# Neural basis of understanding communicative actions: Changes associated with knowing the actor’s intention and the meanings of the actions

**DOI:** 10.1016/j.neuropsychologia.2016.01.002

**Published:** 2016-01-29

**Authors:** Riikka Möttönen, Harry Farmer, Kate E. Watkins

**Affiliations:** a Department of Experimental Psychology, University of Oxford, South Parks Road, Oxford OX1 3UD, UK; b Centre for Functional Magnetic Resonance Imaging of the Brain (FMRIB), University of Oxford, John Radcliffe Hospital, Oxford OX3 9DU, UK

**Keywords:** Action observation, Mirror neuron system, Mirror neurons, Sign language, Communication, Inferior frontal cortex, Inferior parietal lobule

## Abstract

People can communicate by using hand actions, e.g., signs. Understanding communicative actions requires that the observer knows that the actor has an intention to communicate and the meanings of the actions. Here, we investigated how this prior knowledge affects processing of observed actions. We used functional MRI to determine changes in action processing when non-signers were told that the observed actions are communicative (i.e., signs) and learned the meanings of half of the actions. Processing of hand actions activated the left and right inferior frontal gyrus (IFG, BA 44 and 45) when the communicative intention of the actor was known, even when the meanings of the actions remained unknown. These regions were not active when the observers did not know about the communicative nature of the hand actions. These findings suggest that the left and right IFG play a role in understanding the intention of the actor, but do not process visuospatial features of the communicative actions. Knowing the meanings of the hand actions further enhanced activity in the anterior part of the IFG (BA 45), the inferior parietal lobule and posterior inferior and middle temporal gyri in the left hemisphere. These left-hemisphere language regions could provide a link between meanings and observed actions. In sum, the findings provide evidence for the segregation of the networks involved in the neural processing of visuospatial features of communicative hand actions and those involved in understanding the actor’s intention and the meanings of the actions.

## Highlights

• Participants observed hand actions before and after learning that they are signs.
• Learning-induced changes in brain activity measured using fMRI.
• No activity in mirror neuron system when actions were not known to be communicative.
• Knowing the actor’s intention to communicate activated IFG and IPL.
• Knowing meanings of the actions increased activity in left IFG (BA 45), IPL and MTG.

## 1 Introduction

People communicate with each other using speech and manual movements. Co-speech gestures can be integrated with speech and influence how spoken messages are interpreted (Goldin-Meadow, 1999). Some actions, such as pantomimes and emblems (e.g., “thumbs up”, “thumbs down”), can convey meanings independently of speech (Ekman and Friesen, 1969, McNeil, 2005). Also, manual signs can encode meanings in a similar way to spoken words and be used in effective communication among users of signed languages.

The neural basis of manual communication, i.e., how communicative and meaningful hand actions are processed in the human brain, is still poorly understood (for a review, see Andric and Small, 2012). A vast number of studies has investigated processing of another person’s goal-directed, but non-communicative, hand actions in the mirror neuron system (MNS, also often called the action observation network). This fronto-parietal system has been suggested to support understanding of the intentions of an actor through motor mirroring (Rizzolatti et al., 2001, Rizzolatti and Sinigaglia, 2010, Iacoboni et al., 2005). The key areas of the human MNS are the ventral premotor cortex, inferior frontal gyrus (IFG) and inferior parietal lobule (IPL). The human MNS is bilaterally organized (Aziz-Zadeh et al., 2006, Molensberghs et al., 2012). The left-lateralized language network partly overlaps the MNS. The key language areas, such as the left IFG and the posterior temporal cortex (posterior middle and inferior temporal gyri, MTG/ITG), are activated by spoken language but also by communicative hand actions that convey meanings (Lui et al., 2008, Xu et al., 2009, Andric et al., 2013). Although it is clear that both the MNS and language areas participate in processing of communicative and meaningful hand actions, the factors that drive their recruitment remain unclear.

Successful communication via hand actions requires (1) that the observer is aware of the communicative intent of the actor (i.e., why the actor performed the actions) and (2) that she/he knows the meanings of the actor’s hand movements. Little is known about how these two factors that are important for action understanding modulate the neural processing of observed hand actions. Some previous studies have found differences in the neural processing of meaningful and non-meaningful hand actions (Andric et al., 2013, Husain et al., 2012, Decety et al., 1997). It is, however, unclear whether these differences were due to differences in the visuospatial features, familiarity, communicativeness or meaningfulness of the actions. No previous neuroimaging studies have investigated how processing of hand actions changes when their communicative nature or meaningfulness is learned, i.e., when observers “learn to understand actions”.

Here, we used fMRI to investigate how neural processing of observed hand actions changes in the MNS and language regions when people (1) learn that the actions are communicative and (2) learn to associate meanings with the actions. In the first scanning session, non-signers viewed videos of bimanual hand actions, but did not know that they were communicative (“pre-training session”). This session was followed by training during which participants were told that these hand actions are signs in British Sign Language (BSL) and were taught to associate meanings with half of them. Then, participants were scanned again while viewing actions, some of which had known meanings and for the remainder the meanings were unknown (“post-training session”). First, this experimental design allowed us to determine the brain regions that are involved in the processing of dynamic visuospatial features of the hand actions. These brain areas should be activated in both pre- and post-training sessions. Second, the experimental design allowed us to determine the brain regions that are recruited for the processing of hand actions when they are known to be signs, i.e., when the actor’s intention to communicate is known. These brain regions should be non-active in the pre-training session and actions with both known and unknown meanings should activate these regions in the post-training session. Third, the experimental design allowed us to determine the brain regions that are involved in linking meanings with the actions, i.e., regions that are activated more strongly during observation of known compared to unknown actions in the post-training session.

## 2 Material and methods

### 2.1 Participants

17 right-handed non-signers participated in the study. Data from one subject who did not follow task instructions was excluded. The data of 16 participants (6 males, 25–39 years) were included in the analyses. Participants were naïve to the purpose of the study and had no experience with sign language.

### 2.2 Stimuli

55 videos were used in this experiment, 40 of bimanual hand actions that resembled one-word signs used in BSL, 5 videos of the actor standing still and 10 videos of the actor moving her head or shoulders. All hand actions were bimanual and symmetric, i.e., the left and right hands performed identical mirror movements (see Möttönen et al., 2010). The recorded videos did not include any mouth movements. Aside from this, the recorded hand actions resembled real signs in BSL, although the actor who performed them had no training in sign language. Thus, these hand actions were simplified versions of BSL signs. It was important to use such stimuli in the current study, because we wanted to minimize the likelihood that the participants would guess that the hand actions were communicative (i.e., signs) before training. The meanings of the signs used in the study were nouns (e.g., string, magic, rain, cat, and book) and iconic (i.e., the form of the hand movements was related to their meaning) (see Möttönen et al., 2010). Some videos included a repeated movement. For example, in the sign for “rain” the hands with the fingers splayed move downwards twice. The still videos and those with head or shoulder movements were used in a baseline condition. The 40 videos of hand actions were divided into four sets of 10 actions (A, B, C and D) that were matched for duration. The mean duration of videos was 3.4 s (2.6–4.8 s). An additional 7 videos were used during the practice sessions (outside the scanner).

During functional scans, participants were presented with blocks of videos, each containing 5 hand actions from the same set (A, B, C or D) or 5 baseline videos. Each block of hand actions included 0–2 actions with a double movement, i.e., the same hand/arm movement was repeated in the same location of the space. Each baseline block included 4 still videos and 1 video with a head/shoulder movement, which was either a single movement or a double movement. The participants were asked to detect double movements during all blocks, including action and baseline blocks. The average length of each block was 17.4 s (16.3–18.5 s). Presentation of each block was followed by a fixation cross and the mean length of the fixation cross appearance was 6.6 s (5.6–7.7 s). During each functional scan, 30 blocks were presented (e.g., 10 blocks including videos from set A, 10 blocks including videos from set B and 10 blocks including baseline videos). The order of the blocks was pseudo-randomised.

### 2.3 Procedure

#### 2.3.1 Task

During all functional scans, participants indicated after each block of 5 videos whether they had seen any double movements or not by pressing the response button with their left or right thumb (counter-balanced across participants). This task was practiced outside the scanner using an additional set of stimuli to confirm that each participant understood the task. The purpose of this task was to direct participants’ attention to the features of the hand movements and to reduce the likelihood that the participants would realise that the hand actions are communicative. This task was successfully used in our previous study (Möttönen et al., 2010).

#### 2.3.2 Pre-training session

Before the first scan, participants were told that they would see videos of hand movements and were instructed to focus on detecting repeated i.e. double movements in the videos (see Task). Thus, during the first functional scan, the participants did not know that the hand movements were meaningful signs in BSL. During the pre-training scan, half of the participants were presented with sets A and B and the other half were presented with sets C and D.

#### 2.3.3 Pre-training questionnaire

After the pre-training scan, the participants were taken out of the scanner and told that the presented hand movements were signs in BSL and asked to answer following questions: (1) Did you realise that the hand movements were signs? (2) Did you associate any meanings with the signs?

#### 2.3.4 Training

The participants were trained outside the scanner to associate meanings with half of hand actions they saw in the first session (“Old actions”) and half of a new (previously unseen) set of signs (“New actions”). The trained actions were varied across participants so that half of the participants were trained to associate the meanings of sets A and C, and the other half were trained to associate meanings with sets B and D. The experimenter first demonstrated the hand actions and told the participant their meanings. After this the experimenter repeated the actions and the participant was asked the correct meaning. Most participants learnt the meanings of the 20 trained signs after only 2 repetitions. If the participant was unable to remember the meaning of one or more signs then they were repeated a third time. No participant required more than 3 repetitions and all were 100% accurate on their recall of the meaning by the end of this short training.

#### 2.3.5 Post-training session

After training, participants were scanned twice while observing hand actions. One of the scans included the same sets of hand actions as in the first scanning session (i.e., “Old actions”). In order to control possible effects of familiarity, participants were also scanned while observing new, previously unseen, sets of signs (i.e., “New actions”). The order of the “Old actions” and “New actions” scans was counter-balanced across subjects. In other words, all sets (A, B, C and D) were presented to all participants in these two scans (two sets in each scan). In each scan, the participants knew the meanings of one of the sets and the meanings of the other set were unknown. The task performed by participants during the observation of actions was the same as during the pre-training scan.

#### 2.3.6 Post-training questionnaire

After the post-training scans the participants were asked to answer following questions: (1) Did you think about the meanings of the signs you were trained on as you performed the task? (2) Did you realise that the hand movements that you were not trained on were also signs? (3) Did you associate any meanings with the untrained signs?

### 2.4 MRI

Participants were scanned with a 3T Varian scanner with a multislice gradient-echo EPI sequence system (TE = 30 msec, TR = 3000 msec, flip angle = 87°, FOV = 224 mm^2^, voxel size = 3.5×3.5×3 mm^3^, matrix size = 64×64) at the Oxford FMRIB Centre. Forty-two slices with a thickness of 3 mm and no interslice gap were individually positioned to cover the whole brain with reference to a midsagittal scout image. Three functional scans (each with 240 volumes, lasting ~12 min) were acquired during the experiment (one in the pre-training session and two in the post-training session: new and old).

After the pre-training scan, high-resolution anatomical images were acquired for each participant using a T1-weighted 3-D magnetization-prepared rapid acquisition gradient-echo (MP-RAGE) pulse sequence (TE = 5 ms, TR = 13 ms, TI=200 ms, flip angle=8°, FOV= 256×192, matrix size 256×192). One hundred and sixty slices of 1 mm each were positioned to cover the whole brain. This anatomical image was used to aid co-registration to the MNI standard space (see below).

### 2.5 fMRI analyses

The functional MRI data were analysed using FEAT (FMRI Expert Analysis Tool) Version 5.98 running in FSL (the Functional Magnetic Resonance Imaging of the Brain Centre (FMRIB) Software Library). The images were motion corrected by realignment to the middle volume of each run (Jenkinson et al., 2002), unwarped using a fieldmap (Jenkinson, 2003), spatially smoothed with a Gaussian kernel of 7 mm full-width at half maximum and high-pass filtered with a cutoff of 100 s. BET (Brain Extraction Tool) was used to remove signal from non-brain tissue (Smith, 2002). Participants’ functional images were registered to their anatomical image and to standard MNI (Montreal Neurological Institute) images (Jenkinson and Smith, 2001, Jenkinson et al., 2002).

Time-series statistical analyses were performed using a general linear model with local autocorrelation correction (Woolrich et al., 2001). The model for post-training scans used each of the three blocks (Known Actions, Unknown Actions, Baseline) and the two responses (left hand, right hand) as independent explanatory variables. In the model for pre-training scans there was one variable for signs (All Actions). The motion correction parameters (translations and rotations in *x*, *y* and *z*) and volumes corresponding to head motion outliers in each time-series were included as covariates-of-no-interest in the analyses. Statistical maps were calculated for contrasts between Baseline and Known Actions and between Baseline and Unknown Actions for each participant’s each post-training functional scan (Post Old, Post New) and between All Actions and Baseline for each participant’s pre-training functional scan.

To determine group averages, statistical maps from all 16 participants were fed into a group analysis using FMRIB’s Local Analysis of Mixed Effects (FLAME) Version 6.00 (Woolrich, et al., 2004). Anatomical localization was determined through the use of the Juelich histological probabilistic atlas and the Harvard–Oxford cortical atlas; both of which are part of the FSL software.

To determine the brain areas activated by observation of signs, we calculated the Actions > Baseline contrast for each scan separately (Pre, Post Old, Post New). We also contrasted Post Old and Post New Actions in order to find out whether familiarity with the signs affected activation patterns after training. We also contrasted the first and second post-training scans in order to find out whether the order of these scans affected brain activity. The statistical threshold for these contrasts was set to a cluster-forming threshold of *Z* >3.1, with an extent threshold of *p*<0.05 (corrected). Since no significant differences were found relating to familiarity or order, we combined Post Old and Post New scans in subsequent analyses.

Then, in order to determine brain areas in which activity was changed after training, we calculated Pre > Post and Pre < Post contrasts separately for Known Actions and Unknown Actions. We also contrasted Known and Unknown Actions for both pre and post scans in order to determine how knowing the meanings of the signs affected their processing after training. No clusters passed the extent threshold of *p*<0.05 (corrected) for some of these contrasts, we therefore decided to lower the statistical threshold and report clusters that exceeded 50 voxels in extent (*Z*>3.1) to guard against false positives at this uncorrected level of significance.

To further explore the laterality and activation of the sub-regions of the IFG during action observations before and after training, mean percentage signal changes were calculated in a set of regions of interest (ROI). Anatomical ROIs were defined based on cytoarchitectonic probabilistic maps from the Juelich histological atlas (part of FSL): left BA44, left BA45, right BA44, right BA45. Each ROI comprised voxels present in >30% of subjects. Mean percentage signal changes in these ROIs were calculated for each participant, each condition and each session. First, we used one-sample *t*-tests (two tailed) to test whether each ROI showed significant activity during action observation in the pre-training session and paired t-tests to test whether activity differed between the left and right ROIs. Then, we ran repeated-measures ANOVAs with factors Hemisphere (Left vs. Right), Region (BA44 vs. BA45) and Meaning (Known vs. Unknown) for the post-training session to test whether knowing the meanings of the actions affected activity in the left- and right-hemisphere ROIs differently.

## 3 Results

### 3.1 Behavioural results

Twelve of the 16 (75%) participants reported that they had not realised that the hand actions were signs before training. The remaining four participants (25%) reported that they had thought that some of the actions could be signs. However, none of the participants associated any meanings with the actions before training. After training, all participants reported that they had thought about the meanings of trained hand actions during the scans. All participants had also realised that the untrained actions were also signs. Thirteen (81%) of the participants had tried to guess meanings of some untrained actions.

The error rates for detection of the double movements were low in all scans (Pre-training: M=8.75%, SEM: ±2.93; Post-training Old: M=9.17%, SEM=±3.74; and Post-training New: M=10.21%, SEM=±3.77%). This confirms that the participants paid attention to the actions in all scans. There were no differences in task performance across scans (*F*(1,15) < 1, *p*=0.70)

### 3.2 Whole-brain analyses

**Fig. 1:**
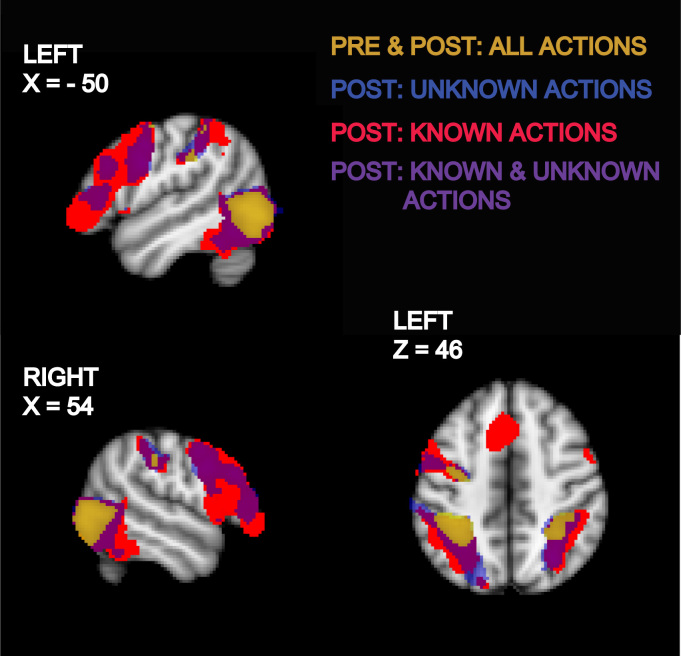
Brain activity during observation of actions in pre- and post-training sessions. Coloured statistical maps representing the group activation for the contrast of observation of actions compared to baseline were thresholded (cluster-forming threshold *Z*>3.1, extent threshold *p*<0.05, corrected) and overlaid on the MNI-152 T1-weighted image. Dark yellow areas were activated during observation of actions in both pre-training and post-training sessions. Blue and red clusters were activated during observation of unknown and known actions respectively in the post-training session. These are coloured purple where they spatially overlap. The coordinate for each slice in MNI-152 standard space is given in mm from the origin. (For interpretation of the references to color in this figure legend, the reader is referred to the web version of this article.)

In the pre-training session, observation of actions activated the lateral occipital cortex and the superior parietal lobule (SPL) bilaterally, and the dorsal premotor cortex in the left hemisphere (Fig. 1, Supplementary Table 1).

In the post-training session, when all participants knew about the communicative nature of the hand actions, observation of action activated more extensive portions of the frontal, parietal and temporal lobes compared to the pre-training session (Fig. 1, Supplementary Table 1). The IFG became active bilaterally after training regardless of whether the meanings of the observed actions were known or unknown. In the parietal lobes, the activation restricted to the SPL pre-training extended towards the intra-parietal sulcus (IPS) and inferior parietal lobule (IPL), and, in the temporal lobes, the posterior parts of the MTG and ITG were activated during action observation after training.

**Fig. 2:**
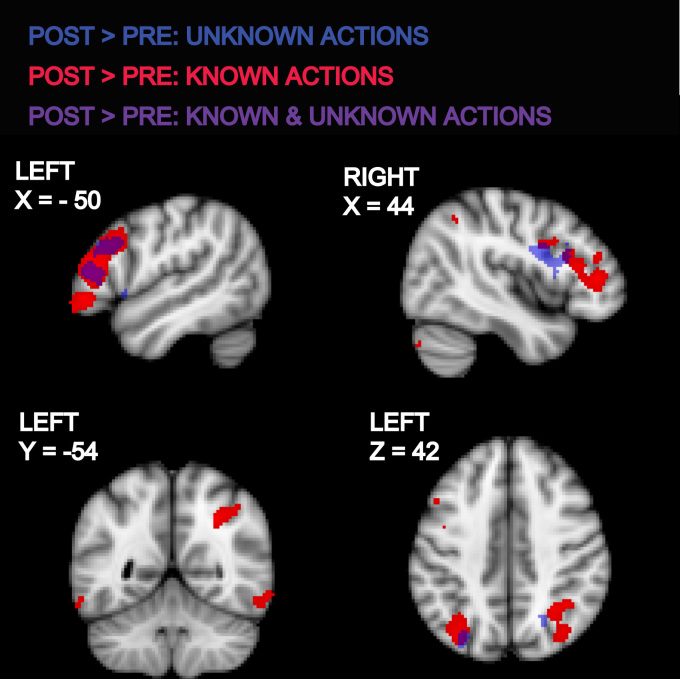
Brain areas showing increased activity when participants knew about the communicative nature of the actions in the post-training session. Coloured statistical maps representing the group activation for the contrast of observing hand actions post- vs. pre-training were thresholded (cluster-forming threshold *Z*>3.1, extent threshold>50 voxels, uncorrected) and overlaid on the MNI-152 T1-weighted image. Blue and red clusters were activated during observation of unknown and known actions respectively to a significantly greater extent in the post-training compared to pre-training session. These are coloured purple where they spatially overlap. The coordinate for each slice in MNI-152 standard space is given in mm from the origin. (For interpretation of the references to color in this figure legend, the reader is referred to the web version of this article.)

When contrasting pre- and post-training sessions, we found increased activity during observation of Unknown Actions (relative to the pre-training session) in the IFG (BA 44 and 45) and in the SPL bilaterally (Fig. 2, Supplementary Table 2). Observation of Known Actions also increased activity in these regions (relative to the pre-training session) but to a greater extent. In addition, observation of Known Actions increased activity in the IPL and posterior ITG/MTG regions bilaterally (Fig. 2, Supplementary Table 2).

**Fig. 3:**
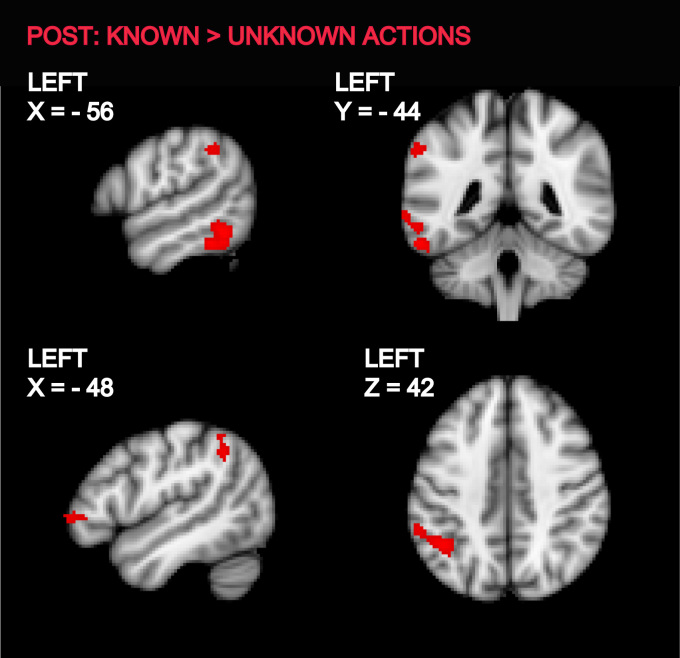
Brain areas showing increased activity when participants were observing actions with known meaning compared to actions with unknown meaning in the post-training session. Statistical maps representing the group activation for the contrast of observing hand actions with known vs. unknown meanings were thresholded (cluster-forming threshold *Z*>3.1, extent threshold>50 voxels, uncorrected) and overlaid on the MNI-152 T1-weighted image. The coordinate for each slice in MNI-152 standard space is given in mm from the origin.

Comparison of activations elicited by Known and Unknown Actions in the post-training session revealed that IFG (BA 45), IPL and posterior ITG/MTG regions in the left hemisphere were more active during observation of Known Actions than Unknown Actions (Fig. 3., Supplementary Table 3).

### 3.3 Anatomical ROI analyses

**Fig. 4:**
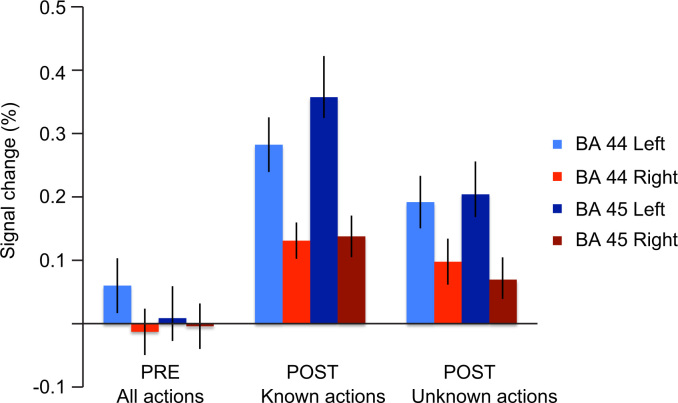
Mean signal changes in the IFG (± standard error, *n*=16). The graph represents mean percentage signal changes in the sub regions of IFG, BA44 and BA45, in the left and right hemisphere during observation of all actions in the pre-training session and actions with known and unknown meanings in the post-training session.

The ROI analysis confirmed that BA 44 and 45 were not activated by action observation in the pre-training session, although they showed robust activity in the post-training session (Fig. 4). The signal changes for action observation (relative to baseline) each participant are presented in Supplementary Fig. 1. The participants who reported that they had guessed that some hand actions could be signs during the pre-training session showed a similar increase in signal changes from the pre- to post-training session as those participants who reported that they had not guessed that that hand actions could be communicative.

ANOVA with factors Hemisphere (Left vs. Right), Region (BA 44 vs. BA 45) and Meaning (Known vs. Unknown) for the percentage signal changes in the post-training session showed significant main effects of Hemisphere (F(1,15) = 11.37, *p*<0.01) and Meaning (F(1,15) = 11.63, *p*<0.01). Thus, the IFG was more strongly activated in the left than right hemisphere, and observation of known actions elicited stronger activation than observation of unknown actions. Meaning also interacted with both Hemisphere (F(1,15)=6.69, *p*<0.05) and Region (F(1,15)=9.77, *p*<0.01). The signal changes for known actions were greater in the BA 45 than BA 44 in the left hemisphere (*p*<0.05), but not in the right hemisphere. The signal changes for unknown actions did not differ between IFG regions in either hemisphere.

## 4 Discussion

In the present fMRI study, we investigated neural processing of communicative hand actions. We presented actions to non-signers before and after telling them that the actions were communicative (i.e., signs) and teaching the meanings of half of the actions. The findings dissociate neural substrates for (i) encoding of dynamic visuospatial features of actions, (ii) processing their communicative nature (i.e., actor’s intention) and (iii) linking the meanings with the actions.

The majority of the participants (75%) did not realise that the observed hand actions were communicative, i.e., signs, in the pre-training session. Although some participants (25%) reported that they realised that some of the actions could be signs, we did not exclude their data from the analyses, because they did not associate any meanings with the actions. In the post-training session, all participants knew that the actions were signs and they associated meanings with the trained actions. Overall, there was a clear difference in the level of awareness of the communicative nature and meaningfulness of the actions between pre- and post-training sessions. Accordingly, the signal changes elicited by observation of actions were greater in the post-training than pre-training session in the IFG even in those participants who had guessed that some of the actions might be communicative.

In the pre-training session, the observation of actions activated the lateral occipital cortex and SPL bilaterally, and the dorsal premotor cortex in the left hemisphere. These activations are likely to reflect processing of low-level features of the hand actions, biological movement and postures. Interestingly, no classic MNS regions (i.e., IFG, IPL, and ventral premotor cortex) were activated during action observation in the pre-training session, although they showed robust activity in the post-training session. Our findings show that observed (non-goal directed) actions are not automatically processed in the MNS when their intention and meaning are not known. This is in agreement with earlier findings showing that meaningless non-goal directed hand actions do not activate the human MNS as strongly as goal-directed actions (Agnew et al., 2012).

The left and right IFG (BA 44 and 45) became involved in sign processing in the post-training session, even when the meanings of the observed actions remained unknown. This pattern of activity is consistent with previous neuromagnetic and neuroimaging studies, which report activity in the left and right IFG during sign observation in non-signers (Levänen et al., 2001, MacSweeney et al., 2004). In these studies, observers also knew that they were observing communicative hand actions. Previous neuroimaging studies have also shown that the IFG is activated by various communicative signals, including audiovisual speech (Miller and D’Esposito, 2005, Ojanen et al., 2005), emblems (Lotze et al., 2006, Villarreal et al., 2008) and co-speech gestures (Dick et al., 2009, Green et al., 2009, Kircher et al., 2009).

The IFG comprises at least two sub-divisions purported to have different functional roles in action and language processing. The anterior IFG, i.e. BA 45, is thought to support semantic processing (Gold et al., 2006, Gough et al., 2005, Hickok and Poeppel, 2007). On the other hand, the posterior IFG, i.e. BA 44, is considered to be the key area of human MNS (Kilner et al. 2009) and to support multiple motor and language functions, such as speech production and phonological processing (Gough et al., 2005). Consistent with this functional division, we found that activity in the left BA 45, specifically, was greater for known than unknown actions, which is likely to reflect retrieval of lexical information, i.e., the newly learned meanings of known actions, from semantic memory. In the current study, both BA 44 and 45 bilaterally were engaged in action processing when their communicative nature was known in the post-training session and actions with either known or unknown meanings activated BA44 to the same extent. Thus, the current findings suggest that both the left and right IFG are involved in processing of communicative actions, in agreement with the bilateral organization of the MNS (Aziz-Zadeh et al., 2006, Molensberghs et al., 2012), whereas processing of the semantic content of actions specifically engages the left IFG (BA 45), in agreement with the typical left-lateralization of the language system.

The MNS is thought to support understanding of “the motor goals and intentions of other individuals” (Rizzolatti and Sinigaglia, 2010). We propose that in the present study, knowing the communicative intent of the actor (i.e., why she performed the actions) led to activation of the mirror neurons in the IFG and IPL/IPS bilaterally in the post-training session. Thus, the results can be interpreted to support the proposal that the MNS plays a role in communication and especially in understanding of the actor’s intentions (Rizzolatti and Arbib, 1998, Rizzolatti and Craighero, 2004, Rizzolatti and Fogassi, 2014). Only a few previous neuroimaging studies have investigated processing of intentions during action observation in humans (for a review see, Rizzolatti and Fogassi, 2014). Iacoboni et al. (2005) found increased activity in the right IFG during observation of grasping actions that were performed in the context that was necessary to understand the intention of the actor (i.e., to clean or to drink). This was seen as the first evidence that the MNS codes intentions in humans. In the current study the intention of the actor was more abstract (i.e., to communicate) and the actions were non-goal-directed. Although we suggest that the bilateral activation of MNS in the post-training session was mainly caused by understanding the actor’s intentions, there are also other processes that may have contributed to the increased activity when actions were known to be communicative. For example, most participants reported that they had tried to guess the meanings of some actions whose meaning they did not know during the post-training session.

Previously, we investigated learning-induced changes in the excitability of motor cortex during observation of communicative actions using a similar experimental design as in the current study (Möttönen et al., 2010). Using transcranial magnetic stimulation, we found that excitability of the left and right motor cortex was not lateralized during observation of bimanual actions while observers were unaware of their communicative nature. After training motor excitability was increased in the left, but not right, motor cortex. The motor excitability became left-lateralized during observation of all actions, that is, both those with known and unknown meanings. We concluded, therefore, that awareness of the communicative nature of the actions enhanced action processing in the left motor cortex of the observers, which was possibly modulated by the IFG. It has been previously shown that the left IFG, especially BA 44, modulates excitability of the left motor cortex during listening to speech (Watkins and Paus, 2004). In the current study we found enhanced activity in the BA 44 and 45 bilaterally for actions with both known and unknown meanings (relative to pre-training), and left-lateralized enhancement in the BA 45 for trained actions (relative to untrained actions). Thus, our findings provide further support for the view that BA 44, but not BA 45, is functionally connected with the left motor cortex during observation of communicative signals. It should be also noted that in the TMS study we found that the observation of signs increased motor excitability in both left and right motor cortex in the pre-training session, whereas no IFG activation was found in the current study before training. This suggests that sensorimotor resonance can increase during observation of hand movements even when the IFG is not activated.

The parietal lobes were activated bilaterally by viewing signs in both sessions. In the pre-training session, the SPL was activated bilaterally, whereas in the post-training session the activity extended into the anterior portion of the IPS and the IPL. The activity in these parietal areas was significantly increased post-training relative to the pre-training session. Moreover, the left IPL was activated more strongly during observation of actions with known than unknown meanings. This pattern of activity in the parietal lobes suggests that the SPL processes visuospatial features of actions (e.g., postures), whereas the IPL and anterior IPS contribute to understanding the intention of the actor. These inferior parietal areas are key areas in the MNS and, in monkeys, they are sensitive to the goals of grasping actions and the motor intentions of an actor (Fogassi et al., 2005). Also, meaningful actions have been shown to activate these regions in humans (Villarreal et al., 2008, Xu et al., 2009).

Our findings also highlight the role of the posterior MTG/ITG region in the processing of meaningful actions. This region was not activated during action observation in the pre-training session and showed enhanced activity in both hemispheres in the post-training session (relative to the pre-training session) for known actions, but not for unknown actions. Moreover, in the post-training session, this region in the left hemisphere was more strongly activated by known compared to unknown actions. It is likely that posterior MTG/ITG region is involved in linking communicative signals with their meanings. According to a recent meta-analysis the semantic system includes, for example, posterior MTG, posterior ITG, IPL and IFG in the left hemisphere (Binder et al., 2009). The posterior ITG/MTG is considered to be a heteromodal area that is activated by both visual and auditory signals and that is involved in supramodal integration and concept retrieval (Binder et al., 2009). Neuroimaging studies on speech comprehension have found activity in this region during lexical-semantic processing (Binder et al., 1997, Rodd et al., 2005, Zhuang et al., 2014), and lesions in this region lead to problems in comprehension of spoken words (Dronkers et al., 2004). Acquisition of word meanings activates both posterior MTG and anterior IFG (BA 45) in the left hemisphere (Mestres-Misse et al., 2008). According to modern neuroanatomical models of speech perception the ventral stream - connecting posterior MTG with IFG - supports speech comprehension (e.g., Hickok and Poeppel, 2007). Our findings suggest that this same pathway also supports comprehension of manual communicative actions.

Observation of sign language has been shown to activate the IFG (BA 44 and 45) in deaf and hearing signers (Neville et al., 1998, Petitto et al., 2000, MacSweeney et al., 2002). Interestingly, however, some studies have shown that observation of pantomimes and simple signs activates the posterior IFG (i.e., the key node of the MNS) in non-signers, but not in signers (Emmorey et al., 2010). Furthermore, damage to these frontal MNS regions in the left hemisphere does not lead to problems in comprehension of signs in deaf signers (Rogalsky et al., 2013). These findings suggest that regions outside the MNS are critical for comprehension of signs in deaf signers, who have life-long experience in manual communication. The lack of activation of the MNS in signers could be explained by the possibility that they automatically link signs with meanings without processing them as actions.

Previous studies have also found activity in the superior temporal cortex during observation of signs in signers (Neville et al., 1998, Petitto et al., 2000, MacSweeney et al., 2002). We did not find such activity in either pre- or post-training session. This may be due to fact that the participants of the current study were hearing non-signers, who had no expertise in BSL. Another possible explanation for this lack of superior temporal activity may be that the signs presented in the current study did not include features that are processed in this area. Our stimulus material consisted of hand actions that resembled signs in BSL performed by a non-signer, whereas natural signs performed by expert BSL signers (and in other signed languages) include also facial expressions and speech-like mouth movements. It is possible that the superior temporal cortex is sensitive to these speech-like movements or other features of natural signs. This hypothesis is supported by a study which showed that in deaf signers the superior temporal regions are activated more strongly by signs with speech-like mouth movements than by manual-only signs (Capek et al., 2008b). Also, in hearing people, viewing speech-related mouth movements activates the superior temporal cortex (Calvert et al., 1997, Capek et al., 2008a, Möttönen et al., 2002). In the current study it was crucial to use manual only, although slightly unnatural, signs, because we wanted to minimize the possibility that the participants would guess that the presented hand actions were signs in the pre-training session.

### 4.1 Summary and conclusions

The findings of the present study highlight the role of prior knowledge in processing of communicative actions. The first aim of the present study was to determine the brain areas that process visuospatial features of communicative actions. These areas (occipital cortex, SPL, and dorsal premotor cortex) were activated when the communicative nature of the actions was unknown. Importantly, the key nodes of MNS (IFG, IPL/IPS, ventral premotor cortex) or language pathways were not activated when the participants did not know about the actor’s intention to communicate. The second aim of the study was to determine how knowing that the hand actions are communicative changes processing of the hand actions. The results showed that this enhanced activity in the IFG (BA 44 and 45) bilaterally, suggesting that the IFG – one of the key areas of the MNS and language networks – is involved in processing of abstract features of hand actions and is possibly involved in understanding the intention of the actor. The other key nodes of the MNS (i.e., IPL/IPS, ventral premotor cortex) were also activated during action observation when the actor’s intention was known. The third aim of the present study was to find out how knowing the meanings of the actions affects their processing. The results showed that the anterior IFG (BA 45), IPL and posterior MTG/ITG in the left hemisphere were more strongly activated during observation of actions with known than unknown meanings. These areas most likely support the linking of meanings with observed hand actions.

Overall, the findings are in agreement with the dual-pathway model of action understanding (Kilner, 2011), which suggest that action understanding is supported by both dorsal (linking IPL/IPS with BA44) and ventral (linking ITG/MTG with BA45) pathways. In the current study, dorsal pathway (i.e., the MNS) showed enhanced activity when the intention of the actor was known, whereas the ventral pathway showed enhanced activity when also the meanings of the actions were known. Thus, processing of actions in these pathways was enhanced by prior knowledge that was essential for action understanding.

## Footnotes

Appendix A Supplementary data associated with this article can be found in the online version at doi:10.1016/j.neuropsychologia.2016.01.002.
