# Supplementary material for: Neural basis of understanding communicative actions: Changes associated with knowing the actor’s intention and the meanings of the actions
Source: Neuropsychologia. 2016 Jan 29;81:230–7. doi: 10.1016/j.neuropsychologia.2016.01.002 (PMC4749541; doi:10.1016/j.neuropsychologia.2016.01.002)
Supplement: Supplementary file 1 — Supplementary material [file mmc1.doc]

**Supplementary Table 1: Peak activations during observation of actions pre- and post-training**

|  | **PRE** |  |  |  |  | **POST KNOWN** | |  |  |  | **POST UNKNOWN** | | |  |  |
| --- | --- | --- | --- | --- | --- | --- | --- | --- | --- | --- | --- | --- | --- | --- | --- |
|  | **Voxels** | **Z-value** | **x** | **y** | **z** | **Voxels** | **Z-value** | **x** | **y** | **z** | **Voxels** | **Z-value** | **x** | **y** | **z** |
| **Left Lateral Occipital Cortex** | **2478** |  |  |  |  | **4443** |  |  |  |  | **7888*** |  |  |  |  |
| Extrastriate visual cortex |  | 4.86 | -36 | -90 | -4 |  | 4.96 | -34 | -94 | -4 |  | 5.35 | -30 | -96 | -8 |
|  |  | 5.17 | -44 | -76 | -10 |  | 5.05 | -42 | -74 | -12 |  | 5.47 | -48 | -72 | 0 |
|  |  | 5.14 | -48 | -74 | -4 |  | 5.14 | -48 | -66 | -4 |  | 5.46 | -38 | -64 | 2 |
| Middle temporal gyrus |  |  |  |  |  |  | 5.11 | -58 | -52 | -4 |  |  |  |  |  |
| Inferior temporal gyrus |  |  |  |  |  |  | 4.83 | -50 | -48 | -22 |  |  |  |  |  |
|  |  |  |  |  |  |  |  |  |  |  |  |  |  |  |  |
| **Right Lateral Occipital Cortex** | **2325** |  |  |  |  | **5113** |  |  |  |  | **4010** |  |  |  |  |
| Extrastriate visual cortex |  | 4.5 | 30 | -98 | -2 |  |  |  |  |  |  |  |  |  |  |
|  |  | 4.14 | 28 | -90 | -8 |  | 5.3 | 34 | -72 | -8 |  | 4.76 | 36 | -84 | -4 |
|  |  | 5.99 | 52 | -68 | -2 |  | 6 | 50 | -66 | -2 |  | 5.78 | 54 | -68 | -2 |
|  |  | 4.95 | 42 | -66 | -8 |  |  |  |  |  |  | 5.22 | 44 | -64 | 4 |
|  |  |  |  |  |  |  | 4.8 | 38 | -64 | -8 |  | 5.03 | 38 | -64 | -8 |
| Cerebellum |  |  |  |  |  |  | 4.79 | 26 | -66 | -34 |  |  |  |  |  |
|  |  |  |  |  |  |  |  |  |  |  |  |  |  |  |  |
| **Left Parietal Cortex** | **1773** |  |  |  |  | **3522** |  |  |  |  | *peaks from cluster above | | | | |
| Superior parietal lobule |  | 4.99 | -32 | -62 | 62 |  |  |  |  |  |  | 5.3 | -26 | -72 | 40 |
|  |  | 4.37 | -36 | -58 | 64 |  | 5.33 | -40 | -46 | 62 |  | 5.69 | -40 | -48 | 60 |
|  |  | 4.76 | -36 | -52 | 58 |  | 5.24 | -40 | -42 | 66 |  | 5.26 | -40 | -42 | 66 |
| Anterior intraparietal sulcus |  | 4.39 | -34 | -46 | 48 |  | 4.88 | -30 | -66 | 34 |  |  |  |  |  |
|  |  | 4.57 | -30 | -40 | 44 |  | 4.88 | -26 | -52 | 48 |  |  |  |  |  |
|  |  | 4.69 | -34 | -38 | 42 |  | 5.13 | -28 | -50 | 42 |  |  |  |  |  |
|  |  |  |  |  |  |  | 5.19 | -40 | -44 | 46 |  |  |  |  |  |
|  |  |  |  |  |  |  |  |  |  |  |  |  |  |  |  |
| **Right Parietal Cortex** | **589** |  |  |  |  | **1877** |  |  |  |  | **1809** |  |  |  |  |
| Superior parietal lobule |  |  |  |  |  |  | 4.94 | 32 | -66 | 50 |  | 5.2 | 30 | -64 | 50 |
|  |  | 3.65 | 30 | -50 | 66 |  | 5 | 34 | -56 | 54 |  | 4.94 | 36 | -52 | 64 |
|  |  | 4.83 | 36 | -48 | 54 |  | 5.58 | 42 | -52 | 60 |  | 4.8 | 32 | -48 | 52 |
|  |  |  |  |  |  |  | 5.36 | 38 | -46 | 56 |  | 4.68 | 30 | -46 | 46 |
| Anterior intraparietal sulcus |  |  |  |  |  |  | 3.28 | 30 | -56 | 30 |  | 5.72 | 32 | -58 | 54 |
|  |  | 3.23 | 42 | -38 | 46 |  |  |  |  |  |  | 3.74 | 28 | -38 | 34 |
|  |  | 3.2 | 42 | -36 | 42 |  |  |  |  |  |  |  |  |  |  |
|  |  |  |  |  |  |  |  |  |  |  |  |  |  |  |  |
| **Left Lateral Frontal Cortex** | **427** |  |  |  |  | **5090** |  |  |  |  | **2575** |  |  |  |  |
| Premotor Cortex BA6 (dorsal) |  | 3.66 | -18 | -12 | 54 |  |  |  |  |  |  |  |  |  |  |
|  |  | 4.26 | -36 | -10 | 52 |  |  |  |  |  |  | 4.76 | -32 | -8 | 50 |
| Premotor Cortex BA6 (ventral) |  |  |  |  |  |  | 5.29 | -46 | -2 | 44 |  | 5.55 | -44 | -2 | 34 |
|  |  |  |  |  |  |  | 4.9 | -44 | 0 | 36 |  | 4.98 | -48 | 0 | 42 |
| Inferior frontal cortex BA44 |  |  |  |  |  |  | 4.6 | -62 | 14 | 2 |  |  |  |  |  |
| Inferior frontal cortex BA45 |  |  |  |  |  |  | 4.73 | -56 | 32 | -2 |  | 4.25 | -54 | 32 | 0 |
|  |  |  |  |  |  |  | 5.01 | -46 | 38 | -6 |  | 4.31 | -52 | 24 | 30 |
|  |  |  |  |  |  |  | 4.96 | -46 | 40 | -10 |  |  |  |  |  |
| Insular Cortex |  |  |  |  |  |  |  |  |  |  |  | 4.24 | -36 | 20 | -4 |
|  |  |  |  |  |  |  |  |  |  |  |  |  |  |  |  |
| **Right Lateral Frontal Cortex** |  |  |  |  |  | **1503** |  |  |  |  | **697** |  |  |  |  |
| Primary Motor Cortex |  |  |  |  |  |  |  |  |  |  |  | 3.65 | 54 | -4 | 34 |
|  |  |  |  |  |  |  |  |  |  |  |  | 3.81 | 50 | -2 | 28 |
| Premotor Cortex BA6 (ventral) |  |  |  |  |  |  |  |  |  |  |  | 3.56 | 56 | 0 | 42 |
| Inferior frontal cortex BA44 |  |  |  |  |  |  | 4.04 | 56 | 4 | 42 |  | 4 | 58 | 6 | 32 |
|  |  |  |  |  |  |  | 4.19 | 42 | 8 | 26 |  | 4.09 | 54 | 8 | 40 |
|  |  |  |  |  |  |  | 4.23 | 54 | 10 | 34 |  |  |  |  |  |
| Inferior frontal cortex BA45 |  |  |  |  |  |  | 4.18 | 50 | 28 | 24 |  |  |  |  |  |
|  |  |  |  |  |  |  | 4.16 | 48 | 32 | 18 |  |  |  |  |  |
|  |  |  |  |  |  |  | 4.04 | 52 | 44 | 12 |  |  |  |  |  |
|  |  |  |  |  |  |  |  |  |  |  | **278** |  |  |  |  |
| Premotor Cortex BA6 (dorsal) |  |  |  |  |  |  |  |  |  |  |  | 4.62 | 38 | -4 | 60 |
|  |  |  |  |  |  |  |  |  |  |  |  |  |  |  |  |
| **Right Medial Frontal Cortex** |  |  |  |  |  | **772** |  |  |  |  |  |  |  |  |  |
| Premotor Cortex BA6 |  |  |  |  |  |  | 4.84 | -2 | 12 | 50 |  |  |  |  |  |
|  |  |  |  |  |  |  | 4.82 | -4 | 18 | 50 |  |  |  |  |  |
| Superior Frontal Cortex |  |  |  |  |  |  | 4.62 | -8 | 20 | 44 |  |  |  |  |  |
|  |  |  |  |  |  |  | 4.38 | -2 | 26 | 46 |  |  |  |  |  |
|  |  |  |  |  |  |  |  |  |  |  |  |  |  |  |  |

**Supplementary Table 2: Peaks of regions activated more during post- relative to pre-training observation of actions**

|  | **POST > PRE KNOWN** | | |  |  | **POST > PRE UNKNOWN** | | |  |  |
| --- | --- | --- | --- | --- | --- | --- | --- | --- | --- | --- |
|  | **Voxels** | **Z-value** | **x** | **y** | **z** | **Voxels** | **Z-value** | **x** | **y** | **z** |
| **Left Parietal Cortex** | **355** |  |  |  |  | **149** |  |  |  |  |
| Posterior parietal cortex |  | 3.28 | -36 | -76 | 50 |  |  |  |  |  |
|  |  | 4.22 | -32 | -68 | 36 |  | 4.18 | -30 | -68 | 38 |
|  |  |  |  |  |  |  |  |  |  |  |
| **Right Parietal Cortex** | **313** |  |  |  |  | **66** |  |  |  |  |
| Posterior parietal cortex |  | 3.55 | 36 | -70 | 42 |  | 3.4 | 12 | -72 | 46 |
|  |  | 3.84 | 38 | -68 | 34 |  |  |  |  |  |
|  |  | 3.46 | 34 | -66 | 46 |  | 3.37 | 22 | -66 | 44 |
| Anterior intra-parietal sulcus |  | 4.11 | 28 | -54 | 38 |  |  |  |  |  |
|  |  | 3.92 | 38 | -52 | 40 |  |  |  |  |  |
|  |  |  |  |  |  |  |  |  |  |  |
| **Cerebellum** | **112** |  |  |  |  |  |  |  |  |  |
| Right anterior lobe |  | 3.29 | 44 | -76 | -36 |  |  |  |  |  |
|  |  | 3.5 | 32 | -74 | -34 |  |  |  |  |  |
|  |  | 3.41 | 30 | -70 | -36 |  |  |  |  |  |
|  |  |  |  |  |  |  |  |  |  |  |
| **Left Posterior Temporal Cortex** | **66** |  |  |  |  |  |  |  |  |  |
| Middle temporal gyrus |  | 3.8 | -60 | -52 | -10 |  |  |  |  |  |
|  |  | 3.44 | -62 | -52 | -2 |  |  |  |  |  |
|  |  | 3.42 | -60 | -48 | -2 |  |  |  |  |  |
|  |  |  |  |  |  |  |  |  |  |  |
| **Right Posterior Temporal Cortex** | **119** |  |  |  |  |  |  |  |  |  |
| Inferior temporal gyrus |  | 3.64 | 60 | -50 | -14 |  |  |  |  |  |
|  |  |  |  |  |  |  |  |  |  |  |
| **Left Lateral Frontal Cortex** | **1661** |  |  |  |  | **411** |  |  |  |  |
| Inferior frontal cortex BA 44 |  | 4.27 | -40 | 16 | 24 |  | 3.43 | -40 | 12 | 28 |
|  |  | 3.45 | -60 | 18 | 12 |  | 3.87 | -40 | 20 | 28 |
| Inferior frontal cortex BA 45 |  | 4.02 | -48 | 28 | 24 |  | 3.93 | -50 | 28 | 28 |
|  |  | 5.06 | -52 | 32 | 10 |  |  |  |  |  |
|  |  | 4.17 | -48 | 38 | -6 |  |  |  |  |  |
|  |  |  |  |  |  | **304** |  |  |  |  |
| Anterior insular cortex |  |  |  |  |  |  | 3.34 | -38 | 16 | -2 |
| Inferior frontal cortex BA 44 |  |  |  |  |  |  | 3.39 | -54 | 20 | 4 |
| Inferior frontal cortex BA 45 |  |  |  |  |  |  | 3.44 | -58 | 28 | 4 |
|  |  |  |  |  |  |  | 4.72 | -52 | 32 | 12 |
|  |  |  |  |  |  | **54** |  |  |  |  |
| Frontal pole |  |  |  |  |  |  | 3.64 | -34 | 60 | 2 |
|  |  |  |  |  |  |  |  |  |  |  |
| **Right Lateral Frontal Cortex** | **734** |  |  |  |  | **395** |  |  |  |  |
| Inferior frontal cortex BA 44 |  | 4.29 | 40 | 2 | 32 |  | 4.3 | 44 | 4 | 24 |
|  |  |  |  |  |  |  | 3.65 | 46 | 12 | 10 |
|  |  |  |  |  |  |  | 3.35 | 32 | 16 | 26 |
| Inferior frontal cortex BA 45 |  | 3.91 | 46 | 28 | 14 |  | 3.68 | 42 | 18 | 20 |
|  |  | 4.1 | 50 | 32 | 8 |  | 3.42 | 48 | 22 | 32 |
|  |  | 4.23 | 50 | 36 | 6 |  | 3.41 | 38 | 24 | 18 |
|  |  | 4.18 | 48 | 40 | 8 |  |  |  |  |  |
|  |  | 4.14 | 42 | 40 | 6 |  |  |  |  |  |
|  |  |  |  |  |  | **68** |  |  |  |  |
| Anterior insular cortex |  |  |  |  |  |  | 3.59 | 32 | 20 | -2 |
|  |  |  |  |  |  | **52** |  |  |  |  |
| Anterior insular cortex |  |  |  |  |  |  | 3.8 | 34 | 0 | 2 |
|  |  |  |  |  |  |  | 3.13 | 34 | 8 | -4 |

**Supplementary Table 3: Peaks of regions that were activated more during observation of actions** with known compared to unknown meaning post-training

|  | **POST KNOWN > POST UNKNOWN** | | | | |
| --- | --- | --- | --- | --- | --- |
|  | **Voxels** | **Z-value** | **x** | **y** | **z** |
| **Left Parietal Cortex** | **272** |  |  |  |  |
| Anterior intraparietal sulcus |  | 3.36 | -40 | -56 | 32 |
| Inferior partietal lobule |  | 3.9 | -42 | -54 | 42 |
|  |  | 3.34 | -46 | -50 | 54 |
|  |  | 3.52 | -58 | -42 | 40 |
|  |  |  |  |  |  |
| **Left Posterior Temporal Cortex** | **350** |  |  |  |  |
| Inferior temporal gyrus |  | 4.26 | -60 | -52 | -14 |
|  |  | 3.86 | -56 | -50 | -20 |
|  |  | 4.03 | -58 | -44 | -20 |
| Middle temporal gyrus |  | 3.41 | -66 | -44 | -2 |
|  |  |  |  |  |  |
| **Left Lateral Frontal Cortex** | **66** |  |  |  |  |
| Inferior frontal cortex BA45 |  | 3.54 | -50 | 46 | 0 |
|  |  | 3.46 | -54 | 38 | -2 |
