# Supplementary material for: Neural basis of understanding communicative actions: Changes associated with knowing the actor’s intention and the meanings of the actions
Source: Neuropsychologia. 2016 Jan 29;81:230–7. doi: 10.1016/j.neuropsychologia.2016.01.002 (PMC4749541; doi:10.1016/j.neuropsychologia.2016.01.002)
Supplement: Supplementary file 2 — Supplementary material [file mmc2.docx]

The figure present percentage signal changes during action observation (relative to baseline) for each 16 subjects in the pre-training and post-training session in four anatomical ROIs (left BA44, right BA44, left BA45, right BA45). Blue and red squares present the signal changes for the actions with unknown and known meanings, respectively. The filled squares show the participants who reported that they had guessed that some actions could be communicative during the pre-training session.
